# Role of Exercise in Fibromyalgia Management: A Narrative Review of Mechanisms, Modalities, and Clinical Evidence

**DOI:** 10.7759/cureus.101299

**Published:** 2026-01-11

**Authors:** Alexander Mazzorana, Laith Fada, John Wahidy, Greg Jacobs

**Affiliations:** 1 Medicine, Alabama College of Osteopathic Medicine, Dothan, USA; 2 Research, Alabama College of Osteopathic Medicine, Dothan, USA

**Keywords:** aerobic training, aquatic therapy, central sensitization, exercise, fibromyalgia, graded activity, pilates, resistance training, tai chi, yoga

## Abstract

Fibromyalgia (FM) is a chronic pain syndrome characterized by widespread pain, fatigue, sleep disturbance, cognitive symptoms, and mood comorbidity. This narrative review synthesizes mechanistic insights, randomized controlled trials (RCTs), and meta-analyses published primarily between 2005 and 2024 to evaluate exercise-based interventions as a primary therapeutic approach for FM. The objective was to synthesize mechanistic rationales, summarize RCTs and meta-analyses across exercise modalities, and provide practical prescription guidance. The results showed aerobic and resistance training consistently reduce pain and improve function and health-related quality of life (HRQoL). Mind-body and aquatic programs were effective and often well tolerated; combined programs frequently yielded the broadest benefits. Serious adverse events were rare. In conclusion, tailored, graded exercise represents an effective, low-risk strategy for FM care. Gaps include long-term durability, response prediction, and dose personalization.

## Introduction and background

Fibromyalgia (FM) is a chronic pain condition that affects about 2%-4% of the adult population worldwide, with prevalence estimates varying by age, sex, and diagnostic criteria [1]. Women are diagnosed approximately two to three times more frequently than men; however, earlier diagnostic frameworks likely underestimated prevalence in men and older adults [2]. It is defined by widespread pain along with fatigue, poor sleep, problems with memory or concentration, and mood symptoms that can greatly affect daily function and quality of life. The diagnosis of FM has changed over time [2]. The 1990 American College of Rheumatology (ACR) criteria relied heavily on tender-point examination, which limited diagnostic sensitivity and contributed to under-recognition in patients without prominent peripheral tenderness. Rather than relying on the traditional tender-point exam, current criteria use symptom-based tools such as the Widespread Pain Index (WPI) and Symptom Severity Scale (SSS). The 2010 and 2011 ACR revisions broadened case identification by incorporating symptom burden and somatic complaints, while the 2016 ACR guidelines also emphasize the importance of “generalized pain” across several body regions, reflecting a shift toward understanding FM as a disorder of pain regulation rather than one of muscle or joint injury [2].

Treating FM can be challenging because no single therapy fully resolves symptoms. Medications such as duloxetine, pregabalin, and amitriptyline can provide partial relief, but their effects are often modest and may come with side effects. For this reason, non-drug therapies have become the main focus of care. The European League Against Rheumatism (EULAR) guidelines identify exercise as the only treatment with a “strong for” recommendation, highlighting it as an essential part of management rather than just an add-on option [3].

Historically, FM research prior to the mid-2000s was limited by inconsistent diagnostic criteria, frequent classification of the condition as psychogenic, and the absence of objective biomarkers, which collectively constrained study design and comparability. As a result, many pre-2005 investigations lack standardized outcome measures and were not included in this review to preserve methodological consistency.

The biology behind FM involves several overlapping systems, including increased central sensitization causing an exaggerated response to pain, reduced pain inhibition, changes in the autonomic nervous system, hormonal imbalances in the hypothalamic-pituitary-adrenal (HPA) axis, and low-grade neuroimmune activation within the central nervous system. Exercise is believed to influence many of these pathways by helping normalize how the brain and spinal cord process pain, improving sleep and energy, reducing stress hormones, and supporting overall physical and emotional health. No validated diagnostic biomarker currently exists for FM; however, investigational biomarkers such as altered cytokine profiles, substance P elevation, and functional neuroimaging patterns continue to be explored [4].

As research continues, exercise is increasingly viewed as a cornerstone of FM care. Developing personalized exercise plans that match each patient’s abilities and symptom levels can improve adherence and outcomes. This review explores the major mechanisms, types of exercise, and clinical evidence supporting physical activity as a key treatment for FM, with included studies published primarily between 2005 and 2024.

Diagnostic and outcome measures referenced in the cited trials included the WPI and SSS, which are freely available as part of the ACR 2016 FM criteria, and the Fibromyalgia Impact Questionnaire (FIQ/FIQ-R) [5], a validated, copyrighted instrument used in accordance with its licensing terms.

## Review

Mechanistic rationale

The pathophysiology of FM is complex and involves both central and peripheral processes that amplify pain perception. The most widely accepted theory centers on central sensitization, in which the brain and spinal cord become overly responsive to pain signals. This leads to a lower pain threshold and a persistent sense of pain even without clear tissue injury. Neuroimaging studies have shown increased activity in pain-processing regions of the brain, such as the insula and anterior cingulate cortex, supporting this idea [6].

Another key factor is dysfunction in descending inhibitory pathways, which normally suppress pain signals traveling from the spinal cord to the brain. Patients with FM often show lowered serotonin and norepinephrine levels, weakening the pathways that normally suppress pain [7]. This imbalance contributes to continuous pain transmission and heightened sensitivity to normally non-painful stimuli known as allodynia.

Autonomic nervous system changes also play a role. Many patients show signs of sympathetic overactivity and impaired parasympathetic tone, which can lead to sleep disturbances, fatigue, and exercise intolerance. In addition, there is evidence of HPA axis dysregulation, resulting in blunted cortisol responses to stress and altered stress hormone rhythms [8]. These endocrine changes can further increase fatigue and pain sensitivity. These alterations are associated with symptom severity rather than structural pathology, further supporting FM as a centrally mediated pain disorder.

Emerging research also highlights low-grade neuroinflammation and glial cell activation within the central nervous system. Elevated levels of pro-inflammatory cytokines such as interleukin-6 (IL-6) and tumor necrosis factor-alpha (TNF-α) have been linked to pain amplification, fatigue, and mood symptoms [9]. Although these markers are not diagnostically specific, they provide mechanistic insight into symptom perpetuation and therapeutic targets.

Exercise is thought to counteract many of these abnormalities. Regular physical activity enhances endogenous pain inhibition by increasing serotonin and norepinephrine availability and stimulating endorphin release. It also promotes neuroplasticity, helping to recalibrate overactive pain networks [10]. Exercise has been shown to improve autonomic balance, reducing sympathetic dominance while enhancing parasympathetic tone, which can improve sleep and overall energy [11].

From a systemic standpoint, exercise reduces inflammatory cytokines and increases anti-inflammatory markers such as IL-10 [11]. It can also normalize HPA axis function, restoring healthier cortisol patterns. Over time, these adaptations improve both physical and psychological resilience.

Overall, exercise provides a biologically sound intervention that addresses the underlying mechanisms of FM rather than just masking symptoms [12]. Evidence suggests that exercise modulates central pain processing, neuroendocrine function, and inflammatory signaling, with effects extending across neural, hormonal, and inflammatory pathways, which may help explain why consistent, moderate physical activity leads to meaningful symptom improvement in most patients [10, 11, 12].

Aerobic training

Aerobic exercise remains the most consistently supported and extensively studied non-pharmacologic intervention for FM. A great degree of evidence, including multiple Cochrane and systematic reviews, demonstrates that structured aerobic training improves pain, fatigue, mood, physical function, and overall health-related quality of life (HRQoL) [11]. Regular participation in aerobic activity also mitigates deconditioning and improves sleep, both of which are strongly associated with symptom burden in this population [13].

Standard protocols typically include two to three sessions per week, lasting 25-45 minutes at light-to-moderate intensity (approximately 40%-60% of maximal oxygen uptake (VO₂ max) or 50%-70% of maximum heart rate) with gradual progression in duration or workload as tolerated [14]. Low-impact activities such as walking, cycling, aquatic exercise, and elliptical training are preferred to minimize joint and soft tissue stress. Meta-analytic data suggest that the most significant improvements in FIQ scores occur with programs lasting 13 to 24 weeks or longer. [15]

The therapeutic effects of aerobic exercise are multifactorial. Physiologically, regular aerobic training enhances central pain inhibition by stimulating opioid and serotonergic pathways, reducing central sensitization, a core pathophysiologic feature of FM. Repeated aerobic engagement improves autonomic balance by decreasing sympathetic overactivity and increasing parasympathetic tone. It also down-regulates pro-inflammatory cytokines such as IL-6 and TNF-α, implicated in FM-related fatigue and high pain sensitivity [16]. Additionally, aerobic activity enhances mitochondrial efficiency, contributing to higher muscle endurance, while psychosocial benefits such as increased self-efficacy, reduced anxiety, and elevated endorphin levels reinforce symptom relief and adherence [17].

Meta-analyses show that aerobic exercise produces large improvements in pain and physical function, effects greater than most other exercise modalities when adherence is maintained [12]. Adherence to aerobic exercise among individuals with FM is variable but critically affects treatment outcomes. Clinical trials generally report completion rates between 60% and 75% for structured aerobic programs, though adherence declines in unsupervised or home-based settings. Symptom fluctuations, such as pain exacerbations and fatigue, are the primary barriers to adherence. Programs that emphasize gradual progression, adequate pacing, and individualized intensity demonstrate better tolerance and retention. Group-based settings achieve the highest adherence, often exceeding 80%, due to social reinforcement. Current data suggest that combining aerobic exercise with resistance or mind-body modalities, such as yoga, produces greater overall improvement in pain, HRQoL, and psychological outcomes than any single form alone [18].

Resistance training

Resistance training has been supported by multiple systematic reviews and randomized trials as one of the most prominent non-pharmacologic treatments for FM. Studies have shown that progressive strength training implemented twice a week has reduced fatigue, pain, and tender point sensitivity [14]. This training has also improved strength, endurance, and overall daily function, which is often reflected in lower FIQ scores [18].

Most programs that showed promising results began with low- to moderate-intensity training and eventually progressed to higher intensity over eight to 16 weeks. A typical session generally consisted of two to three sets of workouts, each with eight to 12 repetitions at 40%-60% of the one-repetition maximum. They would eventually progress to 70% to 80% of their one-rep maximum [16]. Training was achieved with free weights, resistance bands, and exercise machines. Early guidance was recommended to ensure proper technique and pacing occurred. Individualizing the exercise program to fit your needs was also critical in achieving improved outcomes, as it supported long-term adherence [13].

There were multiple benefits that came along with resistance training. Besides gaining strength, physiologic improvement was also seen in motor unit recruitment, coordination, and overall neuromuscular function. These patients often report more confidence in movement, a greater sense of control over their symptoms, and reduced anxiety/avoidance [19, 20]. Furthermore, resistance training also improves central pain modulation via the increase in beta endorphins and serotonin activity. Metabolic changes like improved insulin sensitivity, reduced IL-6 and TNF-α, and increased mitochondrial efficiency all contribute to lower inflammation and better energy regulation [16].

When compared to aerobic exercise, resistance training falls in a similar line in terms of improvement in pain, fatigue, and quality of life. However, there are also additional benefits in strength and functional performance. The 2024 systematic review from above showed that progressive resistance training programs had improved pain, strength, and FIQ scores when compared to aerobic programs [18]. The greatest benefits were seen in combined exercise programs that merged resistance, aerobic, and flexibility training [13].

Resistance training has been proven to be a safe, effective, and long-term therapy for FM treatment. With proper guidance, individualization, and eventual progression, it strongly supplements other approaches that help rebuild physical confidence in patients.

Stretching and flexibility

Stretching and flexibility for FM yield modest but clinically relevant benefits. Its primary function is enhancing muscle ease of movement, relaxation, and overall comfort, rather than reducing pain [21]. 

A 2024 systematic review reported that stretching “may improve pain, HRQoL, and physical and mental function,” although the strength of evidence remains low because of heterogeneous study designs [21]. Proposed mechanisms include reductions in muscle tension and stiffness, increased local blood flow, and increased parasympathetic activation, all of which play a role in decreasing central sensation, one of the core pathophysiologic features of FM. 

When compared to other modalities, stretching alone has a smaller effect on pain reduction and symptom improvement [14]. However, integrating flexibility work into multimodal programs enhances overall tolerance and adherence. One trial found that combining stretching with aerobic training improved sleep quality and decreased FIQ scores more than aerobic exercise alone [22].

In summary, stretching improves flexibility, recovery, and quality of life but is best utilized as a component of a broader exercise regimen. Its low physical demand makes it particularly suitable for individuals with severe pain or limited mobility, serving as a stepping-stone toward more structured aerobic or strengthening programs.

Mind-body interventions (Tai Chi, yoga, and Pilates)

Mind-body exercise modalities such as Tai Chi, yoga, and Pilates integrate controlled physical movement, breath regulation, and mindfulness to address both the physical and psychological dimensions of FM. These practices reduce sympathetic overactivity, enhance central pain modulation, and improve mood, sleep, and coping ability, offering a uniquely holistic approach. Other mind-body interventions, including Qigong and mindfulness-based stress reduction, have also been explored in FM populations, though the strongest and most consistent evidence currently supports Tai Chi, yoga, and Pilates.

Clinical evidence increasingly supports their effectiveness. A 2024 systematic review found that mind-body therapies significantly improved pain, sleep quality, fatigue, and physical function in adults with FM [23]. Tai Chi, in particular, has demonstrated outcomes comparable or superior to aerobic exercise when practiced for at least 12 weeks, improving FIQ scores, mood, and HRQoL [24]. Yoga interventions have shown consistent reductions in pain intensity, anxiety, and depression, while enhancing flexibility and emotional regulation [14]. Similarly, Pilates programs improve postural control, muscle endurance, and global function, often with results comparable to aerobic and aquatic exercise [16].

The mechanisms underlying these benefits include neuroendocrine modulation, with lowered cortisol and catecholamine levels [4], and improved parasympathetic tone through controlled breathing and meditative focus [23]. These shifts may attenuate central sensitization and inflammatory signalling pathways implicated in FM symptom persistence while reducing perceived exertion and pain [4, 16]. Importantly, the low-impact, self-paced nature of these practices supports higher adherence rates and emotional resilience than traditional resistance or aerobic programs, which can provoke symptom flares.

Network meta-analyses have ranked mind-body interventions among the most effective for enhancing HRQoL and global function in FM [14]. Because they simultaneously target body awareness, stress regulation, and movement, they offer a uniquely comprehensive approach that supports long-term management.

In conclusion, mind-body exercise represents a cornerstone of non-pharmacologic therapy for FM [16]. While resistance training often produces larger short-term reductions in pain intensity, mind-body modalities demonstrate superior sustainability and patient acceptability, with benefits extending to improved mood, sleep, and overall quality of life [25].

Aquatic and combined exercise programs

Aquatic exercise utilizes the buoyancy, warmth, and hydrostatic pressure of water to create a low-impact therapeutic milieu ideal for individuals with FM. This environment minimizes joint and muscle loading, enabling both aerobic and resistance activities with reduced nociceptive input and greater exercise tolerance. Warm water immersion (32-34°C) enhances peripheral vasodilation, decreases muscle spasm, and activates parasympathetic pathways, collectively improving circulation, pain modulation, and autonomic balance. Controlled trials and meta-analyses consistently demonstrate significant reductions in pain and fatigue, along with improvements in sleep, mood, and physical function [13,14,18].

Short-term outcomes (eight to 12 weeks) frequently favor aquatic programs due to rapid symptomatic relief and improved movement confidence, whereas extended land-based resistance regimens tend to produce greater musculoskeletal adaptation and pain reduction beyond six months [16]. Higher adherence rates are observed in aquatic settings, attributed to the enjoyable, socially supportive, and low-pain nature of the exercise environment, which likely mediates superior HRQoL outcomes.

Multicomponent programs combining aerobic, resistance, and flexibility exercises, which are often augmented by education or cognitive-behavioral strategies, yield the most durable and comprehensive benefits. Mechanistically, these regimens target multiple dimensions of FM pathophysiology: aerobic training attenuates central sensitization, resistance training improves muscle strength and mitochondrial efficiency, and flexibility work enhances proprioception and range of motion. Behavioral elements concurrently mitigate kinesiophobia and maladaptive coping [13, 14, 16].

Aquatic and integrated combined programs constitute the most evidence-based, patient-centered approach to FM rehabilitation. When individualized and progressed gradually, they optimize neurophysiological adaptation, sustain functional gains, and improve both physical and psychological well-being.

Across exercise modalities, serious adverse events were rare, with reported effects typically limited to transient symptom flares or delayed-onset muscle soreness that resolved with pacing and gradual progression. Table 1 provides a summary of these exercise modalities.

**Table 1:** Comparative summary of exercise modalities in fibromyalgia, outlining key clinical benefits, underlying mechanisms, adherence considerations, and levels of evidence. Mechanisms summarized from [11-18, 23]. HRQoL, health-related quality of life

Exercise Type	Key Benefits/Outcomes	Core Mechanisms	Adherence & Clinical Notes
Aerobic Training	Reduces pain and fatigue; improves HRQoL, mood, sleep, and physical function; reverses deconditioning.	Enhances central pain inhibition via serotonergic / opioid pathways; lowers IL-6 & TNF-α; improves mitochondrial efficiency and autonomic balance.	Best results with 13-24 weeks at light-to-moderate intensity (two to three times/week); group or aquatic formats enhance adherence; gradual progression prevents flares.
Resistance Training	Decreases pain and fatigue; increases strength, endurance, and function.	Promotes neuromuscular adaptation; activates endogenous opioid pathways; improves insulin sensitivity and microcirculation; reduces IL-6/TNF-α.	Two sessions per week, eight to 16 weeks; gradual load progression and supervision essential; measurable strength gains reinforce adherence.
Stretching/Flexibility	Modest improvement in pain and sleep; enhances mobility and relaxation; useful for warm-up or cool-down.	Reduces muscle tension and sympathetic tone; increases parasympathetic activity and blood flow; aids recovery.	Best used adjunctively; safe for severe pain or low fitness levels; enhances tolerance for other exercise.
Mind-Body (Tai Chi, Yoga, Pilates)	Improves pain, fatigue, sleep, mood, flexibility, and coping; enhances HRQoL.	Lowers cortisol/catecholamines; promotes parasympathetic dominance; enhances central pain modulation and body awareness.	Gentle and self-paced → excellent long-term adherence; ideal for stress-sensitive patients.
Aquatic/Combined Programs	Improves pain, fatigue, sleep, and function; combined aerobic-resistance regimens produce the greatest HRQoL gains.	Warm water reduces muscle spasm and sympathetic drive; buoyancy minimizes joint stress; multimodal design targets pain, strength, and flexibility.	High tolerability and enjoyment → excellent adherence; ideal for severe pain or limited mobility; integration with land training maximizes durability.

Discussion

Exercise represents the most evidence-based and cost-effective non-pharmacologic therapy for FM, addressing both symptom control and underlying pathophysiology. Across modalities, consistent findings demonstrate reductions in pain, fatigue, and psychological distress accompanied by improved physical function and HRQoL. The cumulative evidence from randomized controlled trials (RCTs), systematic reviews, and meta-analyses supports aerobic and resistance training as core interventions, while mind-body and aquatic programs provide complementary, patient-centered alternatives that enhance adherence and long-term sustainability.

The benefits of exercise extend beyond symptom relief to encompass systemic and neural adaptations. Aerobic and resistance training show comparable effect sizes for pain reduction and functional improvement, largely mediated by enhanced central pain inhibition, improved mitochondrial efficiency, and reduced inflammatory signaling [13, 18]. Resistance training confers additional advantages in muscular strength and confidence in movement, while aerobic programs improve cardiorespiratory fitness and fatigue resilience.

Mind-body interventions such as Tai Chi, yoga, and Pilates yield multidimensional gains by reducing anxiety, depression, and sympathetic overactivity while improving coping skills and body awareness [23]. These approaches, although often lower in intensity, achieve strong adherence due to their gentle, self-paced nature and emotional reinforcement.

Aquatic exercise uniquely benefits patients with severe pain or limited mobility. Warm-water immersion reduces mechanical loading and facilitates parasympathetic activation, producing rapid short-term symptom relief. In contrast, long-term improvements in muscle strength and endurance are typically greater with land-based resistance programs [14,16].

The most consistent finding across recent network meta-analyses is that multimodal or combined programs that incorporate aerobic, resistance, flexibility, and sometimes cognitive-behavioral elements tend to achieve the greatest overall improvement in global well-being and HRQoL [16]. Such integrated regimens simultaneously address central sensitization, muscle weakness, deconditioning, and maladaptive coping, which are key dimensions of FM's multifactorial pathophysiology.

Exercise is notably safe for patients with FM when introduced progressively. Adverse events are rare and typically limited to transient muscle soreness or symptom flares, which resolve with pacing adjustments. Supervised programs and individualized load progression minimize dropout and enhance confidence. Group settings, aquatic environments, and mind-body modalities offer additional psychological support that sustains adherence, which is an essential determinant of long-term efficacy. 

Despite robust short-term data, several gaps remain. Heterogeneity in study design (particularly in exercise intensity, duration, and supervision) limits direct comparison across trials. Reporting of adherence, adverse events, and maintenance effects beyond 12 months is inconsistent. Few studies identify patient subgroups most likely to benefit from specific modalities or dosing strategies. Additionally, emerging technologies such as virtual-reality-assisted movement therapy and home-based tele-rehabilitation, while not yet systematically evaluated in FM-specific populations, warrant systematic evaluation. Standardized reporting frameworks for exercise prescription, monitoring, and follow-up would strengthen future meta-analytic synthesis.

Clinicians should view exercise not as an optional adjunct but as a first-line, mechanistically targeted therapy. Prescriptions should emphasize low-to-moderate intensity, gradual progression, and patient preference. Aerobic and resistance exercise can serve as foundational components, while mind-body and aquatic modalities provide sustainable options for those with low tolerance or psychological distress. Incorporating education, pacing strategies, and behavioral reinforcement enhances adherence and empowers self-management.

Future research should address long-term durability, phenotype-guided personalization, and dose optimization to determine the minimal effective frequency and intensity needed to sustain benefit. Integrating physiological (autonomic, inflammatory, and neuroendocrine) and psychosocial biomarkers may enable predictive modeling of individual response. Translational efforts should focus on pragmatic, community-based interventions that preserve real-world applicability.

## Conclusions

Exercise is a safe, low-cost, and biologically rational cornerstone of FM management. Across modalities, it improves pain, fatigue, function, and HRQoL through multifaceted neurobiologic and psychosocial pathways. Aerobic and resistance training remain the most evidence-supported core treatments, while mind-body and aquatic programs enhance accessibility and adherence. Combined and graded approaches provide the most comprehensive benefit. Future studies should refine exercise dosing and identify predictors of sustained response to enable fully personalized rehabilitation strategies for patients living with FM.
